# Deciphering the placental abnormalities associated with somatic cell nuclear transfer at single-nucleus resolution

**DOI:** 10.1093/procel/pwad030

**Published:** 2023-05-19

**Authors:** Liyuan Jiang, Xin Wang, Leyun Wang, Sinan Ma, Yali Ding, Chao Liu, Siqi Wang, Xuan Shao, Ying Zhang, Zhikun Li, Wei Li, Guihai Feng, Qi Zhou

**Affiliations:** College of Life Sciences, Northeast Agricultural University, Harbin 150030, China; State Key Laboratory of Stem Cell and Reproductive Biology, Institute of Zoology, Chinese Academy of Sciences, Beijing 100101, China; Institute for Stem Cell and Regenerative Medicine, Chinese Academy of Sciences, Beijing 100101, China; State Key Laboratory of Stem Cell and Reproductive Biology, Institute of Zoology, Chinese Academy of Sciences, Beijing 100101, China; Institute for Stem Cell and Regenerative Medicine, Chinese Academy of Sciences, Beijing 100101, China; Savaid Medical School, University of Chinese Academy of Sciences, Beijing 100049, China; University of Chinese Academy of Sciences, Beijing 100049, China; State Key Laboratory of Stem Cell and Reproductive Biology, Institute of Zoology, Chinese Academy of Sciences, Beijing 100101, China; Institute for Stem Cell and Regenerative Medicine, Chinese Academy of Sciences, Beijing 100101, China; Beijing Institute for Stem Cell and Regenerative Medicine, Beijing 100101, China; College of Life Sciences, Northeast Agricultural University, Harbin 150030, China; State Key Laboratory of Stem Cell and Reproductive Biology, Institute of Zoology, Chinese Academy of Sciences, Beijing 100101, China; Institute for Stem Cell and Regenerative Medicine, Chinese Academy of Sciences, Beijing 100101, China; State Key Laboratory of Stem Cell and Reproductive Biology, Institute of Zoology, Chinese Academy of Sciences, Beijing 100101, China; Institute for Stem Cell and Regenerative Medicine, Chinese Academy of Sciences, Beijing 100101, China; University of Chinese Academy of Sciences, Beijing 100049, China; State Key Laboratory of Stem Cell and Reproductive Biology, Institute of Zoology, Chinese Academy of Sciences, Beijing 100101, China; Institute for Stem Cell and Regenerative Medicine, Chinese Academy of Sciences, Beijing 100101, China; Beijing Institute for Stem Cell and Regenerative Medicine, Beijing 100101, China; State Key Laboratory of Stem Cell and Reproductive Biology, Institute of Zoology, Chinese Academy of Sciences, Beijing 100101, China; Institute for Stem Cell and Regenerative Medicine, Chinese Academy of Sciences, Beijing 100101, China; Beijing Institute for Stem Cell and Regenerative Medicine, Beijing 100101, China; State Key Laboratory of Stem Cell and Reproductive Biology, Institute of Zoology, Chinese Academy of Sciences, Beijing 100101, China; Institute for Stem Cell and Regenerative Medicine, Chinese Academy of Sciences, Beijing 100101, China; Beijing Institute for Stem Cell and Regenerative Medicine, Beijing 100101, China; State Key Laboratory of Stem Cell and Reproductive Biology, Institute of Zoology, Chinese Academy of Sciences, Beijing 100101, China; Institute for Stem Cell and Regenerative Medicine, Chinese Academy of Sciences, Beijing 100101, China; Beijing Institute for Stem Cell and Regenerative Medicine, Beijing 100101, China; State Key Laboratory of Stem Cell and Reproductive Biology, Institute of Zoology, Chinese Academy of Sciences, Beijing 100101, China; Institute for Stem Cell and Regenerative Medicine, Chinese Academy of Sciences, Beijing 100101, China; Beijing Institute for Stem Cell and Regenerative Medicine, Beijing 100101, China; State Key Laboratory of Stem Cell and Reproductive Biology, Institute of Zoology, Chinese Academy of Sciences, Beijing 100101, China; Institute for Stem Cell and Regenerative Medicine, Chinese Academy of Sciences, Beijing 100101, China; University of Chinese Academy of Sciences, Beijing 100049, China; Beijing Institute for Stem Cell and Regenerative Medicine, Beijing 100101, China; State Key Laboratory of Stem Cell and Reproductive Biology, Institute of Zoology, Chinese Academy of Sciences, Beijing 100101, China; Institute for Stem Cell and Regenerative Medicine, Chinese Academy of Sciences, Beijing 100101, China; Beijing Institute for Stem Cell and Regenerative Medicine, Beijing 100101, China; College of Life Sciences, Northeast Agricultural University, Harbin 150030, China; State Key Laboratory of Stem Cell and Reproductive Biology, Institute of Zoology, Chinese Academy of Sciences, Beijing 100101, China; Institute for Stem Cell and Regenerative Medicine, Chinese Academy of Sciences, Beijing 100101, China; University of Chinese Academy of Sciences, Beijing 100049, China; Beijing Institute for Stem Cell and Regenerative Medicine, Beijing 100101, China

## Abstract

**Graphical Abstract:**
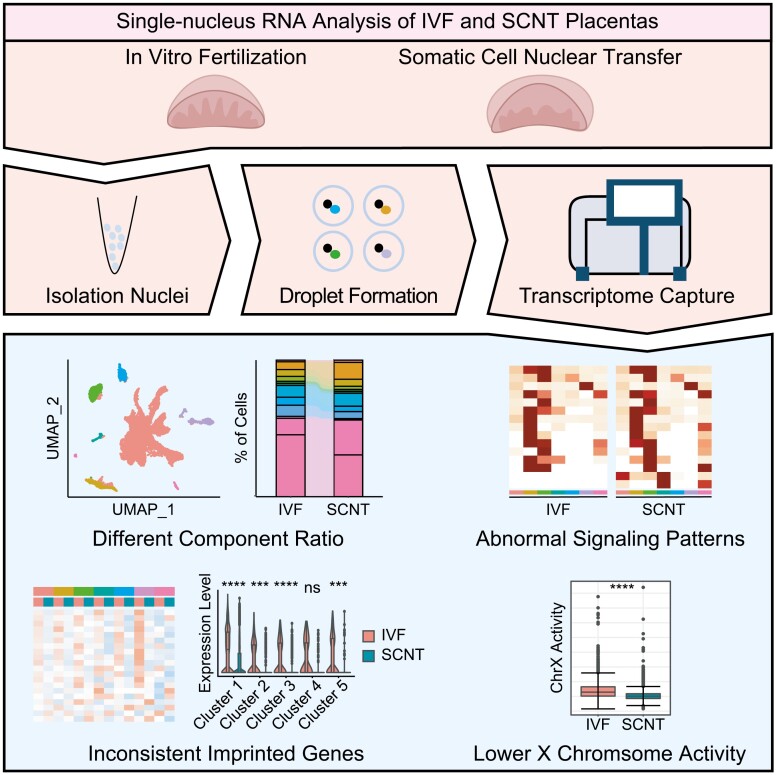

## Dear Editor,

The placenta connecting the fetus to the maternal uterus provides material exchange and an immune-tolerant environment for the embryo in all eutherian mammals (Shao et al., 2022). The representative mouse placenta displays a multilayered structure with distinct characteristics and functions, including the maternal decidua, junctional zone, and labyrinth layer (Marsh and Blelloch, 2020). The decidua, which is thought to be derived from the maternal endometrium (and further undergoes decidualization), provides an anchor for fetal trophoblast invasion. The junctional zone predominantly contains spongiotrophoblasts (SpT), glycogen trophoblasts (GlyT), and trophoblast giant cells (TGCs). The labyrinth is the innermost two-layer structure, which mainly consists of syncytiotrophoblast cells (SynTI and SynTII), sinusoidal TGCs (S-TGCs), and fetal endothelial cells (Simmons and Cross, 2005).

Somatic cell nuclear transfer (SCNT) reprograms a differentiated cell into a totipotent state with great regeneration potential (Matoba and Zhang, 2018). However, the SCNT technique still has some challenges that must be addressed. Previous studies from our laboratory, and other studies, have demonstrated the existence of epigenetic barriers in somatic cell reprogramming. In particular, epigenetic barriers can result in defects in extraembryonic tissues, leading to a low cloning efficiency (Matoba and Zhang, 2018; Matoba et al., 2018; Wang et al., 2020). Large placentas are frequently observed during gestation of cloned animals, regardless of the donor cell type. Although restoring the dosage of some non-canonical imprinted genes could rescue the phenotype of the placenta and enhance cloning efficiency (Wang et al., 2020; Xie et al., 2022), there are still a large number of arrested embryos during E8.5–E11.5 and little is known regarding the changes in cell types or ratio of placentas derived from SCNT. However, it is difficult to investigate the cell composition of the placenta due to the extensive existence of giant or multinucleate cells, where cell size is limited in the sample preparation of 10× single-cell RNA-Seq (scRNA-Seq) (Marsh and Blelloch, 2020). Therefore, no study has analyzed the abnormality of cloned placentas related to wild-type placentas at the single-cell level. The present study addresses this question by establishing a single nuclear RNA transcriptome of SCNT embryos and determining a potential strategy for improving cloning efficiency.

**Figure 1.**
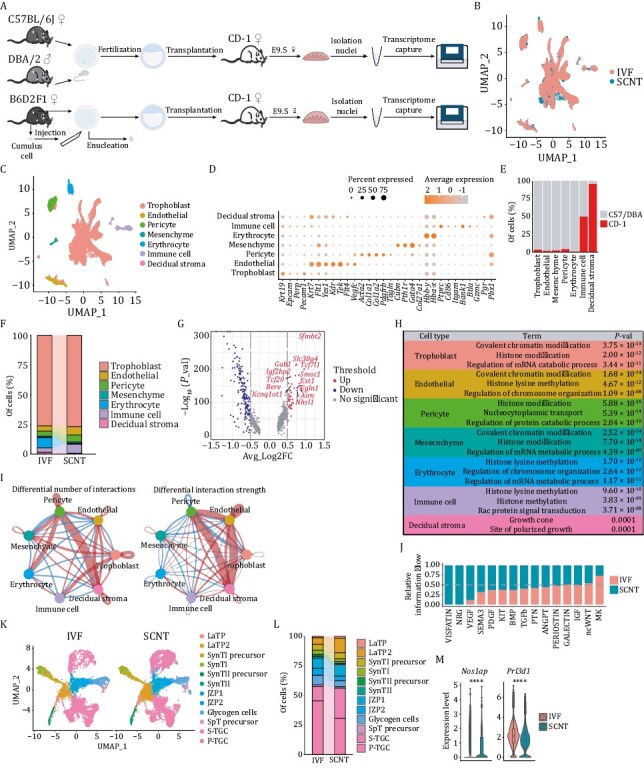
**Single-nucleus atlas of IVF and SCNT embryonic placenta at E9.5.** (A) Schematic diagram of single nuclei harvest procedures from the *in vitro* fertilization (IVF) and somatic cell nuclear transfer (SCNT) groups. (B) Integrated analysis of all nuclei derived from the IVF and SCNT groups through the Uniform Manifold Approximation and Projection (UMAP). (C) UMAP plot of all placental cell types identified by snRNA-Seq analysis. (D) Dot plot of the percentage and average expression level of nuclei in each placental cluster. The analyzed canonical marker genes are listed on the *x*-axis, and the placental clusters are on the *y*-axis. (E) The proportion of each placental cluster derived from different mouse strains based on differential SNP sites. (F) The proportion of different placental clusters in the IVF and SCNT groups. (G) Volcano plot of differentially expressed genes (DEGs) in the IVF and SCNT groups of trophoblasts (up indicates genes with higher expression levels in the SCNT group, down indicates genes with higher expression levels in the IVF group). (H) For each placental cluster, the picked GO terms of highly expressed genes in the SCNT group. (I) Differential interaction number and strength among different clusters in the IVF and SCNT groups (red indicates the number and intensity of increased cell–cell interactions in the SCNT group, and blue indicates the number and intensity of decreased intercellular interactions in the SCNT group). (J) Relative information flow of each signaling pathway from the IVF and SCNT groups. (K) UMAP projection of different subclusters of trophoblast cells in the IVF and SCNT groups. (L) The proportion of different subclusters of trophoblast cells in the IVF and SCNT groups. (M) Violin plot of differential expressions of picked marker genes of S-TGC and P-TGC clusters (*Nos1ap*, *Prl3d1*) in the IVF and SCNT groups. The Wilcox-test was used to statistically test the differential expression of different genes in IVF group and SCNT group samples. **** *P* < 0.0001.

The survival rate of embryos transferred from SCNT was first compared with the survival rate of those transferred from *in vitro* fertilization (IVF). The results showed that only a small proportion of SCNT embryos survived to E9.5 (Table S1), which was in line with our previous findings (Wang et al., 2020). Since the single-nucleus RNA-Seq (snRNA-Seq) can efficiently capture the molecular characterization of complex tissues, regardless of cell size bias, the differences in cellular populations between E9.5-IVF and SCNT embryonic placentas were analyzed by snRNA-Seq to investigate whether placental abnormalities lead to low cloning efficiency (Fig. 1A). After quality control, 6929 and 8443 nuclei were obtained from the IVF and SCNT groups for downstream analysis, respectively. Samples were further integrated to remove batch effects and categorized into seven major cell types annotated by established genetic markers (Marsh and Blelloch, 2020; Zhou et al., 2021). These markers showed distinct expression patterns among specific clusters (Fig. 1B–D). Consistent with the clustering results, strain-specific SNP information showed sequencing reads from the maternal-derived decidual stroma, cell-dominant enriched surrogate mother (CD-1 strain). To our surprise, although fetus-derived immune cells have been reported in the placenta (Chen et al., 2022), we found that partial immune cells came from the fetal side of the placenta at this stage and identified the component ratio of these immune cells to be approximately 50% (Figs. 1E and S1A). Further analysis revealed that the percentages of both immune cells and erythrocytes were significantly different in the placenta from either SCNT or IVF at E9.5, whereas the percentage of trophoblast cells showed no significant difference (Fig. 1F), although our previous study reported the presence of large placentas and increased trophoblast cells in cloned placentas (Wang et al., 2020). Moreover, immunofluorescent staining results verified our sequencing data that the percentage of spongiotrophoblast cells between SCNT and IVF groups has no significant difference (Fig. S1B and S1C). Together, by consideration of the impacts of cell sizes, we used snRNA-Seq analysis to improve and reveal the cellular classification and corresponding proportions of placentas derived from both IVF and SCNT.

Next, the differentially expressed genes (DEGs) for each specific cell type from SCNT and IVF were analyzed. Results showed that chromatin modification-related genes were significantly upregulated in six clusters compared with the gene-expression levels in the corresponding cell clusters in IVF embryos. An exception existed for maternal decidual stroma (Fig. 1G and 1H), which is concurrent with previous knowledge that epigenetic modifications severely influence placental development (Matoba and Zhang, 2018; Matoba et al., 2018; Wang et al., 2020; Xie et al., 2022). Notably, histone methylation-related genes, such as *Ehmt1*, *Ezh2*, and *Setd5*, showed higher expression patterns in all SCNT embryo-derived cells, while some chromatin remodeling-related genes, such as *Ino80* and DNA methyltransferase *Dnmt3b*, which are required to regulate placental development through repression of germline genes (Andrews et al., 2023), exhibited differential expression patterns in certain cell types (Fig. S1D and S1E).

Cell to cell interactions within the fetomaternal interface are undoubtedly crucial for mother–fetus connections. Therefore, we performed intercellular communication analysis and found that decidual stromal cells enhanced interactions with pericytes and endothelial cells (in SCNT placentas) by regulating the cell number and interaction strength (Figs. 1I and S2A). Furthermore, genes involved in the VISFATIN and NRG pathways were upregulated in SCNT cells, suggesting their effects on the interaction difference (Fig. S2B). Among the 15 signaling pathways contributing to major cell communications, the VISFATIN and NRG pathways also mediated the interaction between mesenchymal cells and either trophoblast or decidual stromal cells in the original SCNT sample (Figs. 1J, S2C and S2D).

Trophoblasts are specialized functional cells of the placenta and constitute the largest proportion of our datasets (Fig. 1F). To gain a deeper understanding of trophoblast sub-populations, we performed a subclustering analysis and identified 12 subclusters using specific markers, as previously reported (Simmons and Cross, 2005; Marsh and Blelloch, 2020) (Figs. 1K and S3A). Analysis of DEGs between SCNT and IVF at the subcluster level was also in accordance with the total clustering results. Chromatin modification-related genes were also primarily upregulated in SCNT embryo-derived cell clusters (Fig. S3B and S3C). Notably, we detected trophoblast giant cells (TGCs), which were often missed previously because of their large cell sizes, accounting for nearly 50% of trophoblast cells in our database (Fig. 1L). TGCs were further categorized into two subtypes: parietal-TGCs (P-TGCs) and S-TGCs (Fig. S4A), based on the verified marker gene-expression patterns (Marsh and Blelloch, 2020; Jiang et al., 2023). A higher percentage of S-TGCs was detected in cloned placentas rather than in IVF placentas (Fig. 1L). Reciprocally, the expression levels of marker genes in P-TGCs and S-TGCs also supported these results, regardless of whether the samples were trophoblast cells or whole samples (Figs. 1M and S4B). This result indicated that the cell type difference in TGC could be associated with the functional defects of the cloned placenta.

**Figure 2.**
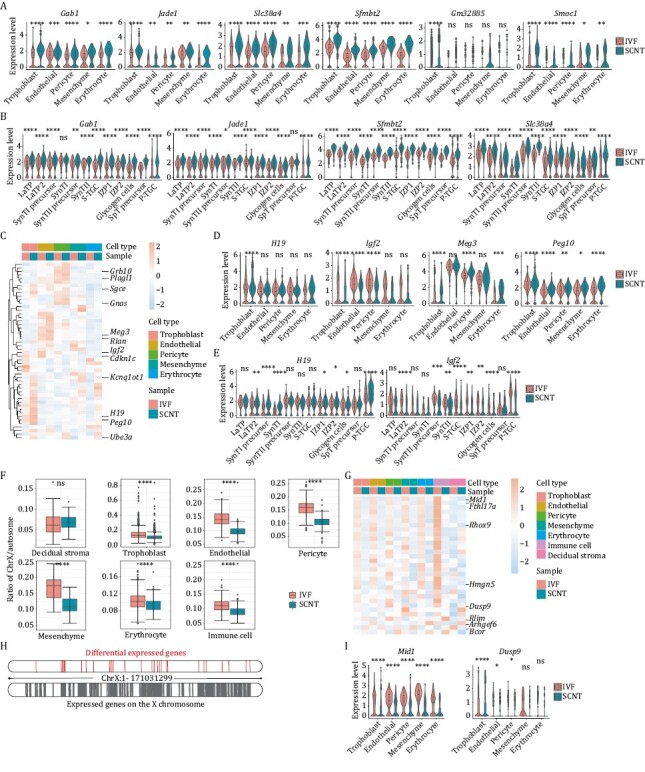
**The expression pattern differences for genes regulated by epigenetic factors of IVF and SCNT embryonic placenta at E9.5.** (A) Violin plot of differentially expressed non-canonical imprinted genes in placental clusters between the IVF and SCNT groups. (B) Violin plot of differentially expressed non-canonical imprinted genes in trophoblast subclusters between the IVF and SCNT groups. (C) Heatmap of differentially expressed canonical imprinted genes in placental clusters between the IVF and SCNT groups. (D) Violin plot of differentially expressed canonical imprinted genes in placental clusters between the IVF and SCNT groups. (E) Violin plot of differentially expressed canonical imprinted genes in trophoblast subclusters between the IVF and SCNT groups. (F) Boxplot of ratio of reads on the X chromosome to reads on autosomes in placental clusters between the IVF and SCNT groups. (G) Heatmap of DEGs on the X chromosome in placental clusters between the IVF and SCNT groups. (H) Location distribution of DEGs on the X chromosome. (I) Violin plot of DEGs on the X chromosome in placental clusters between the IVF and SCNT groups. The Wilcox-test was used to statistically test the differential expression of different genes in IVF group and SCNT group samples. ns means non-significance, * *P* < 0.05, ** *P* < 0.001, *** *P* < 0.001, **** *P* < 0.0001.

Findings from our group, as well as other groups, have shown that epigenetic defects, including non-canonical or canonical imprinted genes and X chromosome inactivation states, severely impede cloning efficiency (Inoue et al., 2010; Wang et al., 2020). However, the underlying cell types affected by epigenetic defects have not yet been clearly explained. Therefore, we first explored the expression patterns of the imprinted genes in different cell types. The snRNA-Seq results showed that the expression levels of non-canonical imprinted genes were heterogeneous and varied among the different cell types (Fig. 2A). Nonetheless, these genes showed stable upregulation in different cell types for SCNT, compared with the corresponding cell types for IVF, especially in trophoblast cells. Similar differences and tendencies were also observed in the sub-populations of trophoblast cells (Figs. 2B and S5A). In contrast to the expression patterns of non-canonical imprinted genes between SCNT and IVF, the expression patterns of several canonical imprinted genes controlled by DNA methylation showed mild differences for certain cell types (Figs. 2C–E and S5B–D). These results indicated several random expression changes of canonical imprinted genes in cloned placentas, which might attenuate developmental efficiency. The detail effects and mechanisms on regulating SCNT efficiency are worthy to study.

Furthermore, to investigate whether abnormal expression of imprinted genes in SCNT was established during reprogramming process, DNA methylation states of imprinting control regions (ICRs) in five different types of cells were analyzed. The results showed that most of ICRs maintained relatively normal methylation patterns in cumulus cells and trophoblast stem cells (TSCs) from IVF groups, while the hyper- or hypo-methylated states were established in SCNT derived TSCs, suggesting these abnormal genes imprinting expression were established during SCNT process (Fig. S5E).

Besides, we examined the X chromosome states in the cloning placenta derived from cumulus cells (Fig. 1A and Table S2), as abnormal inactivation of the X chromosome is another frequently mentioned barrier in SCNT. We computed the ratio of all X-linked reads to all autosomal reads in each cell (Fig. S6A). Interestingly, X chromosome genes were significantly repressed in all types of cloned cells in SCNT samples compared with the corresponding genes in female IVF samples (Fig. 2F). Using the same computing formula, the ratio in the maternal decidual stromal cells did not show any changes, validating the reliability of the database and calculation. The expression pattern of 30 X chromosome genes showed significant changes in the cloned cells, and most of them were downregulated in all cell types for SCNT samples, compared with the gene-expression patterns in the corresponding cell types in IVF samples (Figs. 2G, S6B and S6C). The differential genes were mainly distributed on the X chromosome and were not enriched in some local regions (Fig. 2H). Many of these genes have been reported to be involved in placenta-related developmental processes. *Mid1* has been annotated as a gene that escapes X inactivation, and its mutations have been associated with numerous developmental defects (Baldini et al., 2020). *Dusp9*, which is highly expressed in trophoblast cells, has been reported to be an essential gene for placental development, and is downregulated in some severe pre-eclamptic placentas (Figs. 2I and S6C). In particular, this gene is essential in the labyrinth of the placenta (Czikk et al., 2013). The abnormal expression of *Dusp9* may be associated with the increased cell ratio of LaTP2 and S-TGCs in trophoblasts of SCNT placentas (Fig. 1K and 1L). Together, all these genes could serve as potential targets for enhancing cloning efficiency.

Since X inactive specific transcript (*Xist*) is a critical regulator of X chromosome inactivation, we analyzed the expression pattern of *Xist* in several cell types in SCNT. Results showed that *Xist* was highly expressed in trophoblast, S-TGC and P-TGC subclusters in SCNT group (Fig. S6D). Notably, similar expression pattern of *Xist* has been reported in E10.5 placenta in SCNT from our previous study (Wang et al., 2020). These results indicated that *Xist* expression contributed to X chromosome genes repression in SCNT process. As for the underlying regulatory mechanisms on *Xist* expression in SCNT, we hypothesized that H3K27me3 or H3K27me3 related (de-)methyltransferase might play a role in regulation of X chromosome gene-expression in SCNT based on the results from other researchers that H3K27me3 participates in *Xist* expression (Inoue et al., 2017; Matoba et al., 2018), which is worthy to explore.

Collectively, our study systemically compared the cell compositions of the E9.5 placenta between IVF and SCNT at single-nucleus resolution and explored the potential factors affecting SCNT cloning quality and efficiency. We confirmed that histone modification-related genes dominantly influence various cell types in SCNT placentas. Furthermore, the ratio of S-TGCs to P-TGCs was significantly changed at the subpopulation level. Additionally, we confirmed significant alterations in the expression of non-canonical or canonical imprinted genes and X chromosome genes in SCNT. This finding shed light on how placental defects influence SCNT efficiency. However, more efforts are needed to verify the expression patterns of newly discovered differential targets in SCNT placentas and their impact on improving cloning efficiency.

## Supplementary Material

pwad030_suppl_Supplementary_Materials

## Footnotes

This work was supported by the National Key Research and Development Program of China (2018YFE0201100 to L.W., 2019YFA0110901 to G.F., 2022YFA1104101 to G.F., 2022YFA1104300 to C.L.), the Youth Innovation Promotion Association, CAS (2019084 to G.F.), Informatization Plan of Chinese Academy of Sciences (CAS-WX2021SF-0101 to G.F.) and the National Natural Science Foundation of China (31972895 to L.W.).

The authors declare no competing interests and agree to publish and participate. All animal studies were performed in accordance with the Guidelines for the Use of Animals in Research issued by the Institute of Zoology of the Chinese Academy of Sciences.

The sequencing data were deposited in the Genome Sequence Archive in National Genomics Data Center, China National Center for Bioinformation/Beijing Institute of Genomics, Chinese Academy of Sciences. The accession number for the sequencing data reported in this study is CRA009687.

Q.Z., G.F., L.W., W.L. and Z.L. conceived and designed the study. Q.Z. supervised the project. L.J. and S.M. performed the experiments. X.W., L.J. and Y.D. analyzed the sequencing data. C.L. and X.S. were involved in the methodology. G.F., L.J. and X.W. wrote the manuscript. S.W. and Y.Z. were involved in the manuscript preparation. All authors read and approved the final version of the manuscript. All authors declare no conflict of interests.
