# Supplementary material for: Deciphering the placental abnormalities associated with somatic cell nuclear transfer at single-nucleus resolution
Source: Protein Cell. 2023 May 19;14(12):924–8. doi: 10.1093/procel/pwad030 (PMC10691845; doi:10.1093/procel/pwad030)
Supplement: pwad030_suppl_Supplementary_Materials [file pwad030_suppl_supplementary_materials.docx]

**Supplementary Information**

**Materials and Methods**

**Experimental Animals**

C57BL/6, DBA/2, B6D2F1, and CD-1 mice were purchased from the Beijing Vital River Laboratory Animal Technology Co., Ltd. All animal studies were performed in accordance with the Guidelines for the Use of Animals in Research issued by the Institute of Zoology of the Chinese Academy of Sciences.

**Embryo derivation and culture**

In the IVF group, oocytes from three female C57BL/6 mice were mixed with sperm from male DBA/2 mice in human tubal fluid (HTF) medium and cultured for 4–6 h at 37 ˚C, 5% CO_2_. Fertilized embryos were selected using mouth pipettes and transferred into M16 at 37 °C, 5% CO_2_. The following day, the 2-cell stage embryos were transferred into KSOM and cultured at 37 ˚C, 5% CO_2_. In the SCNT group, cumulus cells and oocytes were separated from female B6D2F1 mice. The cumulus cell nuclei were injected into the enucleated oocytes using a micro-operating system. After activation with SrCl_2_, the embryos were transferred into M16 and cultured at 37 ˚C, 5% CO_2_ overnight. The following day, the 2-cell stage embryos were picked out, transferred into KSOM, and cultured at 37 ˚C, 5% CO_2_.

**Embryo transfer and placental tissue dissection**

Female CD-1 mice were used as pseudo-pregnant surrogate mothers after mating with neutered male mice. Blastula-stage embryos, derived from IVF or SCNT, were transferred into the uterus of E0.5 pseudo-pregnant mice, and the pregnant mice were sacrificed at E9.5 to obtain placentas. Each placenta was separated from the maternal decidua and umbilical cord using forceps, and the remaining placental tissues were placed in precooled PBS to wash off the blood and fragments.

**Sex diagnosis**

In the IVF group, embryonic tissues were collected, and genomes were extracted for sex diagnosis by PCR (Table S2). Female placentas were selected for subsequent examinations.

**Preparation of single nuclei suspensions**

We randomly picked and mixed four placentas per group for single nuclei suspension. The tissues were transferred into new EP tubes containing precooled LB Buffer and then cut into pieces. After lysing on ice for 5–10 min, the solution was passed through a 40 µm filter into a new EP tube and then centrifuged at 500 × g for 5 min at 4 °C. The supernatant was carefully removed, and 300 µL of LB Buffer was added to resuspend the pellet. Thereafter, 300 µL of RB Buffer was added, and the solution was mixed gently. Following this, 600 µL of PB1 Buffer was added from the bottom of the tube with the pipette tip. Thereafter, 600 µL of PB2 Buffer was slowly added from the bottom of the tube, and the mixture was centrifuged at 4000 × g for 20 min at 4 °C. The nuclei layer between PB1 and PB2 was transferred to a new EP tube. The RB Buffer (1 mL) was added, and the pellet was resuspended gently. After passing through a 40 µm filter, the solution was centrifuged at 500 × g for 5 min at 4 °C. Finally, the supernatant was carefully removed, and 100 µL of EB Buffer was added to resuspend the nuclei. Single-nuclei suspensions were used for the subsequent examinations.

**Single nuclei RNA sequencing**

All single-cell nuclei were sorted using FACS Aria II (BD Biosciences, San Jose, CA, USA) using a 70 mm nozzle. Nuclei were sorted according to the steps outlined above (<https://www.protocols.io/view/frankenstein-protocol-for-nuclei-isolation-from-f-3fkgjkw/abstract>). Briefly, single nuclei were suspended in PBS containing 0.04% BSA. Approximately 6,000 nuclei were added to each channel, and the target cell recovered was estimated to have around 3,000 nuclei. Captured nuclei were lysed, and the released RNA was barcoded through reverse transcription in individual GEMs. Reverse transcription was performed using S1000TM Touch Thermal Cycler (Bio-Rad) at 53 °C for 45 min, followed by 85 °C for 5 min, and held at 4 °C. The cDNA was generated and amplified, and the quality was assessed using an Agilent 4200 (performed by Capital Bio Technology, Beijing). Single-nuclei RNA-seq libraries were constructed according to the manufacturer’s instructions using the Single Cell 3 Library and Gel Bead Kit V3.1. Finally, the libraries were sequenced using an Illumina Novaseq 6000 sequencer with a sequencing depth of at least 100,000 reads per nucleus with a pair-end 150 bp (PE150) reading strategy.

**Single nuclei RNA analysis**

The raw data were aligned to the reference genome mm10 using Cell Ranger Single-Cell Software Suite 3.1.0 in include-intron mode, and a barcode-count matrix was generated. Seurat was used for the downstream analysis (Mangiola et al., 2021). Nuclei with fewer than 200 unique genes, greater than 30% ribosomal counts, and all nuclei with greater than 30% mitochondrial counts were excluded from all subsequent analyses. Because of differential hemoglobin counts between the two samples, different parameters were set for different samples (nuclei with greater than 1% of the IVF group samples and greater than 10% of the hemoglobin counts of the SCNT group samples were excluded from all subsequent analyses). Average numbers of 1,909 and 2,144 for the expressed genes per nucleus and 3,088 and 3,488 for counts in each nucleus of the two samples were detected. A batch effect correction was performed between the two groups and integrated into one dataset using Harmony (Korsunsky et al., 2019), the combined data were scaled and normalized, and the default 2,000 highly variable genes were selected by “FindVariableFeatures” and used for future analyses. The first 40 dimensions were selected for the PCA analysis, and dimensionality reduction and visualization were performed using Runumap. “FindAllMarkers” were used to find the marker genes for each cluster. Potential doublets were identified using “Doublet Finder” (McGinnis et al., 2019), after which they were removed, and the remaining nuclei were subjected to the same analysis workflow (scaling, normalization, dimensionality reduction, clustering, etc.). The trophoblast cluster was extracted from the entire dataset, and the same workflow was performed. Different classical cell-type markers were used to annotate the different cell types. Differentially expressed genes between the different cell types were identified using “FindAllMarkers” with the following parameters (min.pct = 0.25, logfc.threshold = 0.25).

**Gene function enrichment analysis**

A gene function analysis was performed on specified differentially expressed gene sets using “ClusterProfiler” (Wu et al., 2021), and the terms with p-value＜0.05 were considered.

**Cell communication analysis**

Cell annotations and cell count matrices of the two groups were used as inputs for Cell Chat analysis (Jin et al., 2021). Cell-to-cell communication analysis between the two groups was performed separately according to the differentially expressed genes in different cell types. Potential cell communication pathways between the different cell types in each group were analyzed.

**Imprinted genes and X chromosome-linked genes analysis**

Genes located on the X chromosome of mm10 and all imprinted genes curated in the study by Santini et al. (Santini et al., 2021) were used for the differential expression analysis.

**Cells from maternal origin analysis**

We used “bcftools” (Danecek and McCarthy, 2017) to call the SNP of CD-1 and C57/DBA mice. We searched for conserved CD-1-specific SNP sites through the whole genome sequencing data (Jung et al., 2022) and the SNP information of different mouse strains published. This information was used to distinguish between maternal or paternal X chromosome inactivation.

**Differential DNA methylation analysis**

We re-analyzed three DNA-methylation seq replicates of cumulus cells samples, nine replicates of IVF-derived TSCs samples and three replicates of SCNT-derived TSCs samples, compared with the methylation levels of oocyte and sperm which were calculated in the previous paper (Sun et al., 2021), and checked them in the 54 ICRs which reported in the previous paper (Ci and Liu, 2015).

**Immunofluorescent** **staining**

Placentas were frozen in liquid nitrogen with OCT (SAKURA, 4583), and then sliced into 10 mm sections. The frozen sections were placed for 30 min and treated with 4% PFA for 10 min at room temperature. After rinsing with PBS, incubated the sections in blocking buffer (5% BSA) for 1 h, and subsequently incubated with the primary antibody (Tpbpα, Abcam, ab104401) for 4 h at room temperature. Washed the slices with PBS for three times, then incubated with secondary antibody (Goat anti-mouse 488, Invitrogen, A11001) Nuclei were stained with Hoechst 33342 (Invitrogen H3570). The images were captured under a tissue multispectral quantitative analysis system (PerkinElmer). Area ratios were measured with Photoshop.

**References**

Ci, W., and Liu, J. (2015). Programming and inheritance of parental DNA methylomes in vertebrates. Physiology (Bethesda) 30, 63-68.

Danecek, P., and McCarthy, S.A. (2017). BCFtools/csq: haplotype-aware variant consequences. Bioinformatics 33, 2037-2039.

Jin, S., Guerrero-Juarez, C.F., Zhang, L., Chang, I., Ramos, R., Kuan, C.H., Myung, P., Plikus, M.V., and Nie, Q. (2021). Inference and analysis of cell-cell communication using CellChat. Nat Commun 12, 1088.

Jung, Y.-H., Wang, H.-L., Ali, S., Corces, V.G., and Kremsky, I. (2022). Chracterization of a strain-specific CD-1 reference genome reveals potential inter- and intra-strain functional variability.

Korsunsky, I., Millard, N., Fan, J., Slowikowski, K., Zhang, F., Wei, K., Baglaenko, Y., Brenner, M., Loh, P.R., and Raychaudhuri, S. (2019). Fast, sensitive and accurate integration of single-cell data with Harmony. Nat Methods 16, 1289-1296.

Mangiola, S., Doyle, M.A., and Papenfuss, A.T. (2021). Interfacing Seurat with the R tidy universe. Bioinformatics 37, 4100-4107.

McGinnis, C.S., Murrow, L.M., and Gartner, Z.J. (2019). DoubletFinder: Doublet Detection in Single-Cell RNA Sequencing Data Using Artificial Nearest Neighbors. Cell Syst 8, 329-337 e324.

Santini, L., Halbritter, F., Titz-Teixeira, F., Suzuki, T., Asami, M., Ma, X., Ramesmayer, J., Lackner, A., Warr, N., Pauler, F.*, et al.* (2021). Genomic imprinting in mouse blastocysts is predominantly associated with H3K27me3. Nat Commun 12, 3804.

Sun, J., Zheng, W., Liu, W., Kou, X., Zhao, Y., Liang, Z., Wang, L., Zhang, Z., Xiao, J., Gao, R.*, et al.* (2021). Differential Transcriptomes and Methylomes of Trophoblast Stem Cells From Naturally-Fertilized and Somatic Cell Nuclear-Transferred Embryos. Front Cell Dev Biol 9, 664178.

Wu, T., Hu, E., Xu, S., Chen, M., Guo, P., Dai, Z., Feng, T., Zhou, L., Tang, W., Zhan, L.*, et al.* (2021). clusterProfiler 4.0: A universal enrichment tool for interpreting omics data. Innovation (Camb) 2, 100141.

**Figure S1. The differentially expressed genes between IVF and SCNT embryonic placenta at E9.5.**

(A) UMAP projection of all nuclei analyzed in inferred parental origin.

(B) Immunofluorescent staining of E9.5 placentas of the SCNT and IVF groups. Spongiotrophoblast cells were stained with *Tpbpα* (green) and DNA was stained with Hoechst 33342 (blue). Scale bars are 500 μm.

(C) Scatter plot of area ratio of spongiotrophoblast cells to the whole IVF or SCNT placenta tissue, respectively. For IVF group, n=3; For SCNT group, n=4.

(D) Multi-volcano plot showing the number of genes differentially expressed in the IVF and SCNT groups for each placental cluster. The numbers at the top indicated genes with higher expression levels in the SCNT group, and the numbers at the bottom indicated genes with higher expression levels in the IVF group.

(E) Violin plot of differentially expressed genes associated with epigenetic modifications between the IVF and SCNT groups for each placental cluster.

The Wilcox-test was used to statistically test the differential expression of different genes in IVF group and SCNT group samples. ns means non-significance, * *P* < 0.05, ** *P* < 0.001, *** *P* < 0.001, **** *P* < 0.0001.

**Figure S2. The cell-to-cell communication analysis for cells from the IVF and SCNT groups.**

(A) Heatmap comparison of overall signaling of all cells between the IVF and SCNT groups.

(B) Circle plot of inferred SCNT-specific intercellular signaling pathways (VISFATIN and NRG signaling pathways).

(C) Violin plot of differentially expressed genes involved in the VISFATIN pathway between the IVF and SCNT groups.

(D) Violin plot of differentially expressed genes involved in the NRG pathway between the IVF and SCNT groups.

The Wilcox-test was used to statistically test the differential expression of different genes in IVF group and SCNT group samples. ns means non-significance, * *P* < 0.05, ** *P* < 0.001, **** *P* < 0.0001.

**Figure S3. The clustering and differentially expressed gene analysis for trophoblast cells.**

(A) Dot plot showing the percent and average expression of nuclei in each trophoblast subcluster. The analyzed canonical marker genes are listed on the x-axis, and the trophoblast subclusters are on the y-axis.

(B) The multi-volcano plot showing the number of genes that were differentially expressed in each trophoblast subcluster between the IVF and SCNT groups (The numbers at the top indicate genes with higher expressions in the SCNT group, and the numbers at the bottom indicated genes with higher expressions in the IVF group).

(C) GO terms of highly expressed genes in the SCNT group for each trophoblast subcluster.

**Figure S4. The marker genes for S-TGC and P-TGC.**

(A) Violin plot of picked marker genes of S-TGC and P-TGC clusters (*Nos1ap*, *Podxl*, *Prl2c2*, *Prl3b1*, *Prl3d1*, *Prl4a1*) for each trophoblast subcluster in the SCNT group.

(B) Violin plot of picked marker genes of S-TGC and P-TGC clusters (*Podxl*, *Prl4a1*, *Prl3b1*) in the IVF and SCNT groups.

The Wilcox-test was used to statistically test the differential expression of different genes in IVF group and SCNT group samples. **** *P* < 0.0001.

**Figure S5. The expression pattern of non-canonical or canonical imprinted genes.**

(A) Violin plot of differentially expressed non-canonical imprinted genes in trophoblast subclusters between the IVF and SCNT groups.

(B) Violin plot of differentially expressed canonical imprinted genes in placental clusters between the IVF and SCNT groups.

(C) Heatmap of differentially expressed classical imprinted genes in trophoblast subclusters between the IVF and SCNT groups.

(D) Violin plot of differentially expressed canonical imprinted genes in trophoblast subclusters between the IVF and SCNT groups.

(E) Heatmap of different methylation levels of the ICRs among oocyte, sperm, cumulus cell, TSCs derived from IVF embryos and TSCs derived from SCNT embryos, respectively.

The Wilcox-test was used to statistically test the differential expression of different genes in IVF group and SCNT group samples. ns means non-significance, * *P* < 0.05, ** *P* < 0.001, *** *P* < 0.001, **** *P* < 0.0001.

**Figure S6. The expression pattern of genes on the X chromosome.**

(A) Boxplot of ratio of reads on the X chromosome to reads on autosomes reads on autosomes in all nuclei analyzed between the IVF and SCNT groups.

(B) Violin plot of differentially expressed genes on the X chromosome in placental clusters between the IVF and SCNT groups.

(C) Violin plot of differentially expressed genes on the X chromosome in trophoblast subclusters between the IVF and SCNT groups.

(D) Violin plot of differentially expressed *Xist* in trophoblast, S-TGC and P-TGC subclusters between the IVF and SCNT groups.

The Wilcox-test was used to statistically test the differential expression of different genes in IVF group and SCNT group samples. ns means non-significance, * *P* < 0.05, ** *P* < 0.001, *** *P* < 0.001, *****P* < 0.0001.

**Table S1.** Development of Embryos from SCNT and IVF.

**Table S2.** Primers for PCR.

| Table S1. Development of Embryos from SCNT and IVF. | | | | |
| --- | --- | --- | --- | --- |
| Groups | Donor Cells | No. of Embryos Transferred | No. of Implantation Sites at E9.5 (% of Transferred Embryos) | No. of E9.5 (% of Transferred Embryos) |
| IVF | - | 145 | 104 (71.7 ± 7.4)^a^ | 70 (48.3 ± 6.7)^b^ |
| SCNT-1 | B6D2F1-cumulus | 120 | 33 (27.5 ± 8.9)^c^ | 2 (1.7 ± 1.2)^d^ |
| SCNT-2 | B6D2F1-cumulus | 160 | 76 (47.5 ± 15.4)^e^ | 8 (5 ± 4.7)^f^ |
| All embryos were transferred at the blastula stage. For a versus c, *P* < 0.01. For a versus e, non-significant difference; For b versus d, *P* < 0.001; For b versus f, *P* < 0.0001. | | | | |

| Table S2. Primers for PCR. | |
| --- | --- |
| Name | Sequence (5’-3’) |
| mus zfy F | AAGATAAGCTTACATAATCACATGGA |
| mus zfy R | CCTATGAAATCCTTTGCTGCACATGT |
| mus phex F | TGAGCAGGTAGGCAGTC |
| mus phex R | GAGGTAAGGCAGGGTC |
